# Femtosecond Laser Processing Technology for Anti-Reflection Surfaces of Hard Materials

**DOI:** 10.3390/mi13071084

**Published:** 2022-07-08

**Authors:** Xiaofan Xie, Yunfei Li, Gong Wang, Zhenxu Bai, Yu Yu, Yulei Wang, Yu Ding, Zhiwei Lu

**Affiliations:** 1 Center for Advanced Laser Technology, Hebei University of Technology, Tianjin 300401, China; xiaofan_xie@126.com (X.X.); baizhenxu@hotmail.com (Z.B.); yuyu1990@hebut.edu.cn (Y.Y.); wyl@hebut.edu.cn (Y.W.); zw_lu@sohu.com (Z.L.); 2 Hebei Key Laboratory of Advanced Laser Technology and Equipment, Tianjin 300401, China; 3 Tianjin Key Laboratory of Electronic Materials and Devices, Tianjin 300401, China; 4 National Demonstration Center for Experimental (Electronic and Communication Engineering) Education, Hebei University of Technology, Tianjin 300401, China; 5 Science and Technology on Electro-Optical Information Security Control Laboratory, Tianjin 300308, China

**Keywords:** femtosecond laser processing, anti-reflection, micro/nanostructures, biomimetic structures, hard materials

## Abstract

The anti-reflection properties of hard material surfaces are of great significance in the fields of infrared imaging, optoelectronic devices, and aerospace. Femtosecond laser processing has drawn a lot of attentions in the field of optics as an innovative, efficient, and green micro-nano processing method. The anti-reflection surface prepared on hard materials by femtosecond laser processing technology has good anti-reflection properties under a broad spectrum with all angles, effectively suppresses reflection, and improves light transmittance/absorption. In this review, the recent advances on femtosecond laser processing of anti-reflection surfaces on hard materials are summarized. The principle of anti-reflection structure and the selection of anti-reflection materials in different applications are elaborated upon. Finally, the limitations and challenges of the current anti-reflection surface are discussed, and the future development trend of the anti-reflection surface are prospected.

## 1. Introduction

The anti-reflection characteristic of material surface is beneficial to improve the ability to distinguish specific electromagnetic signals, and help to shield and eliminate clear interference signals [1]. In window materials, reducing the reflection of incident light on the material surface and improving its transmission is crucial, since higher transmittance can bring higher precision and better stability. In optoelectronic devices, the enhancements of absorption with anti-reflection structures decrease the losses of optical power, resulting in high energy conversion efficiencies [2,3,4,5,6]. Therefore, it is crucial to modulate and manage incident light by fabricating anti-reflection surfaces.

There are kinds of ways to prepare anti-reflection surfaces, including the sol-gel method [7], electron beam etching [8], nano-imprinting [9], wet etching [10], dry etching [11], etc. The sol-gel method is generally employed to prepare porous anti-reflection films, but its preparation time is relatively long. The nano-imprinting method can transfer the micro/nanostructure of the template to the target material by photoresist assistance, with the advantages of simple processing, high speed, and high precision. However, the master mold requires a complex manufacturing process and high cost. Wet etching is a fast and efficient method that is widely used in the preparation of anti-reflection structures, but it is not easy to control the isotropic etching process. In contrast, laser processing technology can realize the arbitrary processing structure with high-precision controllability. Moreover, laser processing technology can be programmable, which is suitable for large-area processing of a bevy of materials [12,13,14,15,16], including metals, semiconductors, carbon-based materials, polymers, etc. [17,18,19,20,21,22,23]. These advantages enable the laser processing technology to predominate in the fields of micro-nano processing such as micro-optics and micro-fluidics [24,25,26,27,28]. In recent years, femtosecond laser processing technology has drawn significant attentions in micro-nano processes performed on hard materials due to its ultra-short pulse duration and ultra-high peak power density [29,30,31,32,33,34]. The negligible thermal effect and high degree of controllable designability is very beneficial to the design and post-production of anti-reflection surfaces. Combined femtosecond laser processing with chemical etching can reduce the surface roughness of the processed materials [35,36,37], resulting in the improvement of the optical properties. Therefore, femtosecond laser processing plays a vital role in the field of preparing anti-reflection surfaces.

This review summarizes the recent advances in the field of anti-reflection surface preparation of hard materials by femtosecond laser, elaborates the principle of the anti-reflection structure, focuses on the preparation method of anti-reflection surfaces on hard materials, and summarizes the applications of anti-reflection surfaces.

## 2. Basic Principles and Fabrication Methods

### 2.1. Basic Principles

When light propagates in a medium, its propagation path will remain unchanged. However, when light is incident on the interface of two media with different refractive indices, the propagation path of light will change. Part of the light will return to the original medium, which is caused by a sudden change in the refractive index (R) at the boundary. In practical applications, this part of the reflected light will cause considerable losses of energy, so reducing the reflected light on the material surface has always been hotly pursued.

Fresnel reflection happens if light is incident from one medium to another one, and the reflection of light at the interface could be described by the usage of the Fresnel equation. The Fresnel equation provides the basic model for traditional anti-reflection coatings, with two assumptions: (1) the reflected waves possess the same intensity, with one wave reflected from each interface; (2) other optical interacting conditions such as scattering and absorption can be ignored [38].

The thin film (refractive index n <$\;n_{s}$) on the substrate ($n_{s}$) basically obeys the law of thin-film interference. The following two conditions must be satisfied for the two reflected waves to interfere destructively: (1) the difference of phase between the reflected waves is π; (2) the thickness (d) of the film have to be λ/4 with an odd number of times, where λ is the wavelength of the incident light. As we known, phase difference δ=2πnd cosθ/λ (θ is the incidence angle), when the light of incidence is incident perpendicular to the interface (i.e., θ=0), the reflectance of the interface R can be expressed as:

(1) $$R={\left(\frac{n_{0}n_{s}-n^{2}}{n_{0}n_{s}+n^{2}}\right)}^{2}\;$$

where $n_{0}$ is the air refractive index. In order to make R=0, from the formula (1), it can be known that $n=\sqrt{n_{0}n_{s}}$, d=λ/4n. Therefore, to achieve the minimum light reflection, it is critical to adjust the thickness of the film as well as the refractive index.

It has been found that some insects, such as moths, have a large number of nano-pillar arrays distributed on the surface of their compound eyes. This compound eye structure eliminates the reflected light on the surface of the moth’s eyes, which is beneficial for moths to observe targets at night and guarantee flight safety [39,40,41,42]. Due to their special optical properties, a slurry of researching work has been inspired by moth eye structures [43,44] to reduce reflectivity by introducing micro/nanostructures. Several typical biological surfaces with anti-reflection as well as other functions are listed in Table 1.

The mechanism of the interaction of the incident light with the anti-reflection structure is briefly described below by taking the inverted cone structure of the bionic moth-eye as an example.

**Figure 1:**
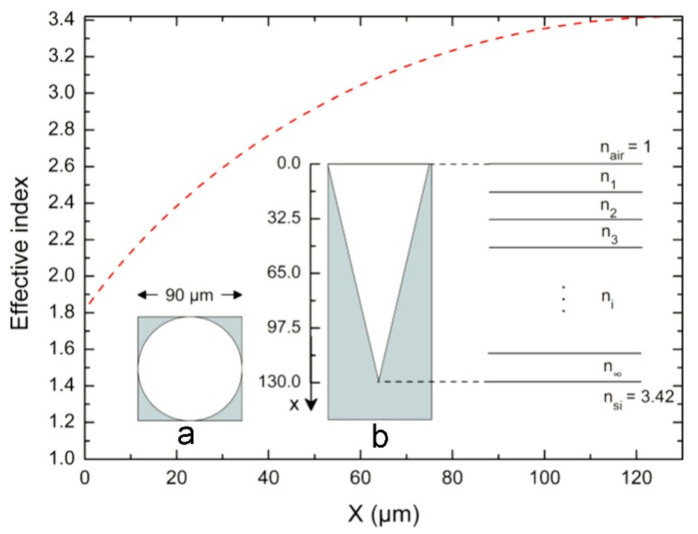
Schematic diagram of multilayer graded index of refraction for a single inverted cone structure and effective index for different vertical distances x, (**a**) Top view and (**b**) longitudinal section [61], Copyright © 2016 Elsevier.

As shown in Figure 1, the inverted cone structure (in this example, the structure period is 90 μm and the depth is 130 μm) can be regarded as combining infinite layers with different refractive indices $n_{i}$, and the effective refractive index $n_{i}$ of each layer can be given by the following formula:

(2) $$n_{eff}=\sqrt{\left(1-\alpha \right)n_{1}^{2}+\alpha n_{2}^{2}}$$

where $n_{1}$ and $n_{2}$ are the refractive indices of air and material, respectively, and α is the fill factor (area fraction) of the material in every layer. If the vertical distance from each layer to the material surface is defined as x, the fill factor α can be expressed as:

(3) $$\alpha =1-\frac{\pi }{4}\times {\left(1-\frac{x}{L}\right)}^{2}$$

where L is the height of the entire inverted cone structure. After calculating the fill factor α for different vertical distances x, the relationship of effective index and vertical distances x can be obtained as shown in Figure 1. It could be explicitly seen that the curve of the effective refracting index keeps increasing with the vertical distance x. According to the Fresnel formula:

(4) $$t=\frac{2n_{2}}{n_{1}+n_{2}}$$

If the refracting index of adjoining layers $n_{1}$ and $n_{2}$ are very close, the transmittance of incident electromagnetic waves is close to 100%.

The mechanism of other anti-reflection structures is similar to that of the inverted cone structure, in which the sudden change of the refractive index of the different media is transformed into a graded refractive index gradient, thereby reducing the reflection of light between the two media [42]. The effectiveness of the anti-reflection structures has been confirmed on hard materials such as silicon, zinc sulfide, and sapphire.

### 2.2. Fabrication Methods

At present, methods for fabricating anti-reflection surfaces are generally divided into two categories: anti-reflection coatings and anti-reflection structures. Anti-reflection coatings introduce one or more thin films on the material surfaces to reduce reflectivity [62]. From $n=\sqrt{n_{0}n_{s}}$ and d=λ/4n, it can be seen that one layer of the film corresponds to one wavelength. If it needs to work under multiple wavelengths or in a wide wavelength range, dozens or even hundreds of films are required to be prepared on the material surface to achieve anti-reflection. Such a fabrication method can greatly aggravate the complexity of the process. In addition, the introduction of multi-layer films leads to poor mechanical stability and low thermal matching, which makes it difficult to meet the requirements for anti-reflection in the full-angle wide spectral range.

Compared with anti-reflection film, the anti-reflection structure has higher mechanical strength and stability. The surface structure is more designable and has the advantages of a large anti-reflection bandwidth and small incident angle dependence. There are many methods to fabricate the anti-reflection structures, such as chemical growth [63], plasma etching [64], photolithography [65], and laser processing. The chemical growth method is easy to operate, low in cost, and good in applicability, but it is difficult to realize anisotropic etching. Laser processing is a very mature processing technology, which possess the advantages of high efficiency, robust design ability, large-area processing, and non-contact. Table 2 lists the detailed fabrication methods, including advantages, disadvantages, and optical properties of various anti-reflection structures on typical hard materials.

## 3. Femtosecond Laser Processing Anti-Reflection Structures

The femtosecond laser has extremely high peak power, with instantaneous power density reaching the order of terawatts [76,77,78]. The ultra-short pulse makes its energy absorption time much smaller than the relaxation time of the thermal effect, resulting in no thermal damage to the processed material. The ultra-high instantaneous power makes it exhibit a nonlinear absorption effect when interacting with materials. Due to multi-photon absorption at the focal point, femtosecond laser processing can break through the diffraction limit of optical processing to realize nano-scale structures. In addition, as a kind of laser direct writing technology, femtosecond laser processing has strong designability and significant advantages in the fabrication of complex three-dimensional structures. Femtosecond laser processing technology has already been proven as one of the means in the field of advanced micro-nano manufacturing and has extensive applications regarding micro-fluidics, micro-optics, and biomedicine [79,80,81].

Femtosecond laser processing technology can be roughly classified into two types: (1) femtosecond laser multi-photon polymerization processing, which is an additive processing technology mainly used for processing polymer materials [82,83]; (2) femtosecond laser ablation processing, which is mainly used to process hard materials [84,85,86,87,88]. As follows, we focus on the femtosecond laser fabrication of anti-reflection structures on the surface of typical hard materials and the related studies on the principles of femtosecond laser ablation processing.

### 3.1. Silicon

**Figure 2:**
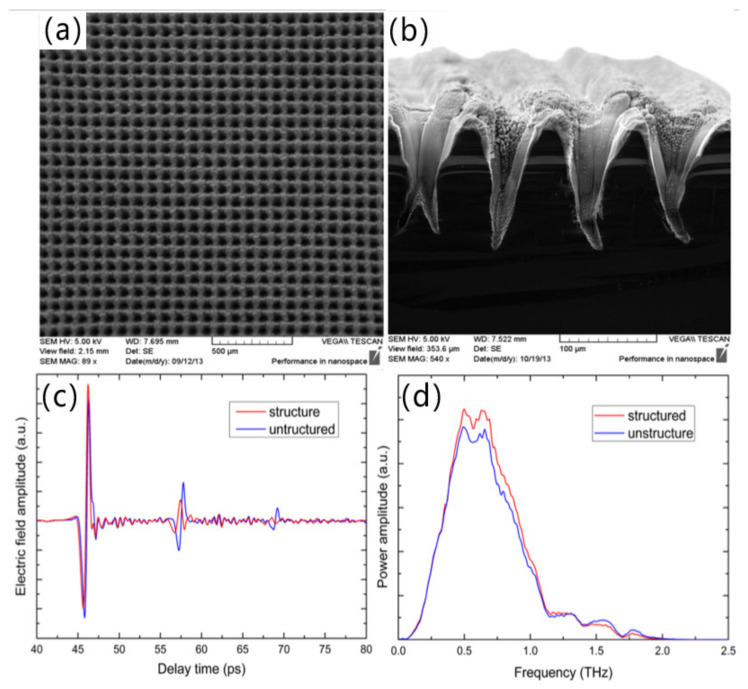
(**a**) Topping view and (**b**) Crossing sectional view of the inverted cone-shaped structure arrays with a period of 90 μm on a high-resistance silicon substrate by SEM; (**c**) Time-domaining signal and (**d**) Frequency-domaining spectrum of inverted conical structure sample [61], Copyright © 2016 Elsevier.

Silicon is a type of essential material used for photovoltaic industry. Due to its large refractive index, light irradiated onto the surface of silicon is not fully absorbed. A large portion of the incident light is reflected, while the light loss due to high reflection on the silicon surface seriously affects the conversion efficiency of photovoltaic devices. Therefore, it is necessary to design structured anti-reflection surfaces on the silicon surface [23,69]. It has been shown that one-dimensional grating structures can significantly improve the optical properties of semiconductor surfaces and effectively suppress reflected light. In 2011, Vorobyev et al. [23] used femtosecond laser pulses to irradiate silicon surfaces, resulting in periodic nano-grating stripe structures on silicon surfaces. Such structured silicon surfaces exhibited very low UV to a near-infrared (200–2500 nm) wide spectral range of reflectance. In 2016, Zhang et al. [61] employed a femtosecond laser with different powers and pulse numbers to fabricate inverted cone-shaped structure arrays with a period of 90 μm on a high-resistance silicon substrate. Compared with unstructured silicon, the transmittance of structured silicon in the range of 0.32~1.30 THz is increased by a maximum of 14%, as shown in Figure 2. It is confirmed that the transmission windows can be tuned by altering the inverted tapered structure with different periods.

**Figure 3:**
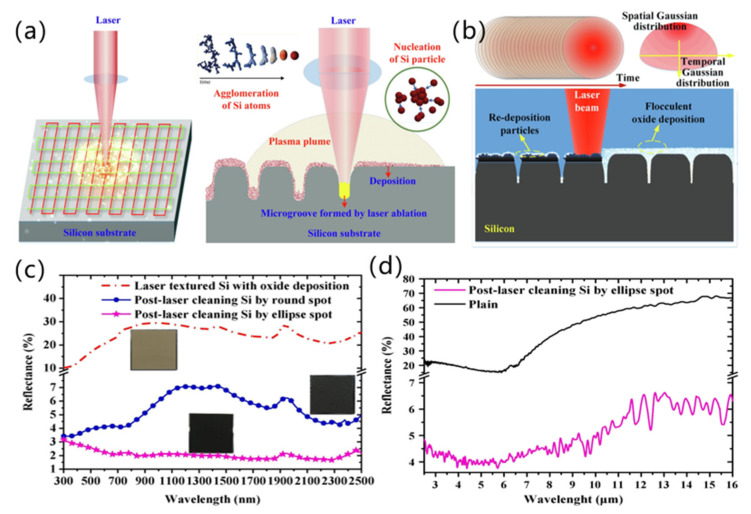
(**a**) Laser ablation kinetics of micro/nanostructure growth paths and deposition on silicon substrates; (**b**) Schematic diagram of round dot laser cleaning oxide deposition; (**c**) Reflectance spectra of textured silicon surfaces in the range of 300–2500 nm; (**d**) Reflection spectra of micro/nano-construction fabricated by laser cleaning assisted laser ablation irradiation and unprocessed silicon within MIR region (2.5–16 μm) [89], Copyright © 2020 Elsevier.

When a laser interacts with silicon, deposited particles are easily generated around the ablation area. These particles and debris are easily oxidized to silicon oxide and deposited on the silicon surface during the processing. This results in a greatly reduced performance of the fabricated anti-reflection structure surface. In 2020, Chen et al. [89] exploited laser cleaning technology to assist femtosecond laser ablation of the silicon surface, which effectively eliminated oxide deposition. The processed products had an average reflectivity of 2.06% in the range of 300 to 2500 nm and 4.3% in the range of 2.5 to 10 μm, and it was confirmed that the surface roughness and reflectivity of silicon obtained by laser cleaning assisted femtosecond laser ablation were lower than those obtained by hydrofluoric acid etching assisted femtosecond laser ablation, as shown in Figure 3.

### 3.2. Metal

Metal plays a key role in realizing the functions in the fields of solar absorbers [90,91], military stealth [92,93], molecular detection [94,95], etc. However, effective reduction of the metal surfacing reflection remains a difficult task. The majority of metals possess large optical constants, leading to massive optical impedance between metal and air [96]. Common coating strategies are usually ineffective in bridging the refractive index gap between the metal surface and air [38,97]. In contrast, the reduction of reflectivity of a metal surface through constructing micro/nanostructures has also gained much attention in recent years [98,99]. Micro/nano-scaled structures have special optical absorption properties, including the geometric trapping effect and surface plasmon resonance, which can effectively reduce the reflectivity of metal surfaces [100,101].

**Figure 4:**
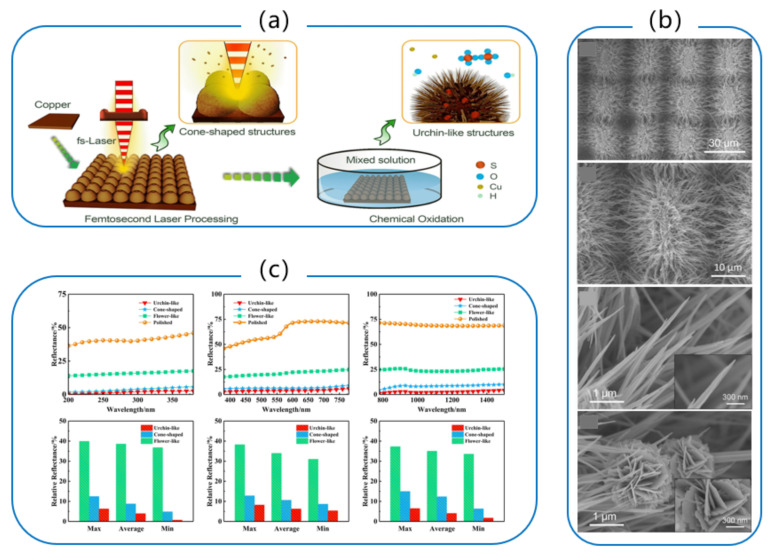
(**a**) The synergetic fabrication process of the urchin-like array; (**b**) SEM images regarding morphological features of urchin-like arrays and individual urchin-like structures; (**c**) Anti-reflection performance of micro/nanostructures in VIS, UV, and IR bands [105], Copyright © 2020 Elsevier.

In 2017, Yao et al. [102] fabricated moth-eye microstructures and nanoparticle-covered laser-induced periodic surface structures (NC-LIPSS) on the surface of 304 stainless steel at different polarizations and energy densities via the femtosecond laser processing technique. The relationship between the size of the micro/nanostructures, laser energy density, and the orientation of NC-LIPSS and laser polarization were demonstrated. The laser-irradiated surface had better anti-reflection properties compared to pristine stainless steel. In 2021, Xu et al. [103] applied the femtosecond laser line scan technique to process parallel microgrooves with a period of 50 μm on the surface of aluminum alloy and obtained an anti-reflection performance of less than 5% in the spectral range of 200 to 1200 nm. In 2018, Fan et al. [104] applied the femtosecond laser direct writing technique into the fabrication of highly disordered super level micro/nanostructures on metallic copper surfaces, achieving an average hemispheric reflectance of 2.4%, 5.5%, and 6% in the wavelength ranges of 400–800 nm, 200–2000 nm, and 2.5–25 μm, respectively, with an average absorption of 94% within the range of 0.2–25 μm. In 2020, Liu et al. [105] proposed a femtosecond laser pretreatment combined with a chemical oxidation growth micro-nano fabrication strategy to prepare sea urchin-like composite micro/nanostructures on the surface of metallic copper, which exhibited excellent reflectance reduction and obtained ultra-low reflectance in an ultra-wide spectrum including UV, visible, IR, and far IR. In the UV band, the mean reflectance of the sea urchin-like array was merely 3.9% of that of polished copper, with a minimum value as low as 0.7%. The minimum reflectance in the visible band reached 5.3% of that of polished copper with a value fluctuation of less than 1%; while the minimum relative reflectance in the IR band was only 1.7%, indicating excellent and stable reflection reduction capability as shown in Figure 4. This chemical oxidation growth-assisted femtosecond laser processing strategy provides a simple method for fabricating micro/nanostructures combined with high homogeneity and reproducibility. The fabricated heterogeneous sea urchin-like structures can be employed as core functional progenitors in photovoltaic equipment, stealth materials, and chemical catalysis.

### 3.3. Sapphire

Sapphire crystals are widely used in defense, industrial, and space optics because of their high hardness, good chemical/thermal stability, and high light transmittance covering the ultraviolet to mid-infrared bands [106]. In the civil field, sapphire is mainly used for wear-resistant structural parts, medical implant materials, high-temperature windows, blue LED substrate materials, laser dielectric materials, optical lenses, watch and mobile phone windows, etc. In the military field, sapphire is used as a variety of infrared window material for equipment such as fairings for infrared-guided missiles, optoelectronic pods for fighter jets, optoelectronic masts for submarines, etc. At the same time, sapphire also has important applications in the aerospace field and can be used as a large-sized space optical window material [107,108,109,110,111,112,113]. However, the large refractive index of sapphire results in relatively high reflectivity at the interface of air/sapphire. The resulting light transmission efficiency of the sapphire surface is not high enough.

**Figure 5:**
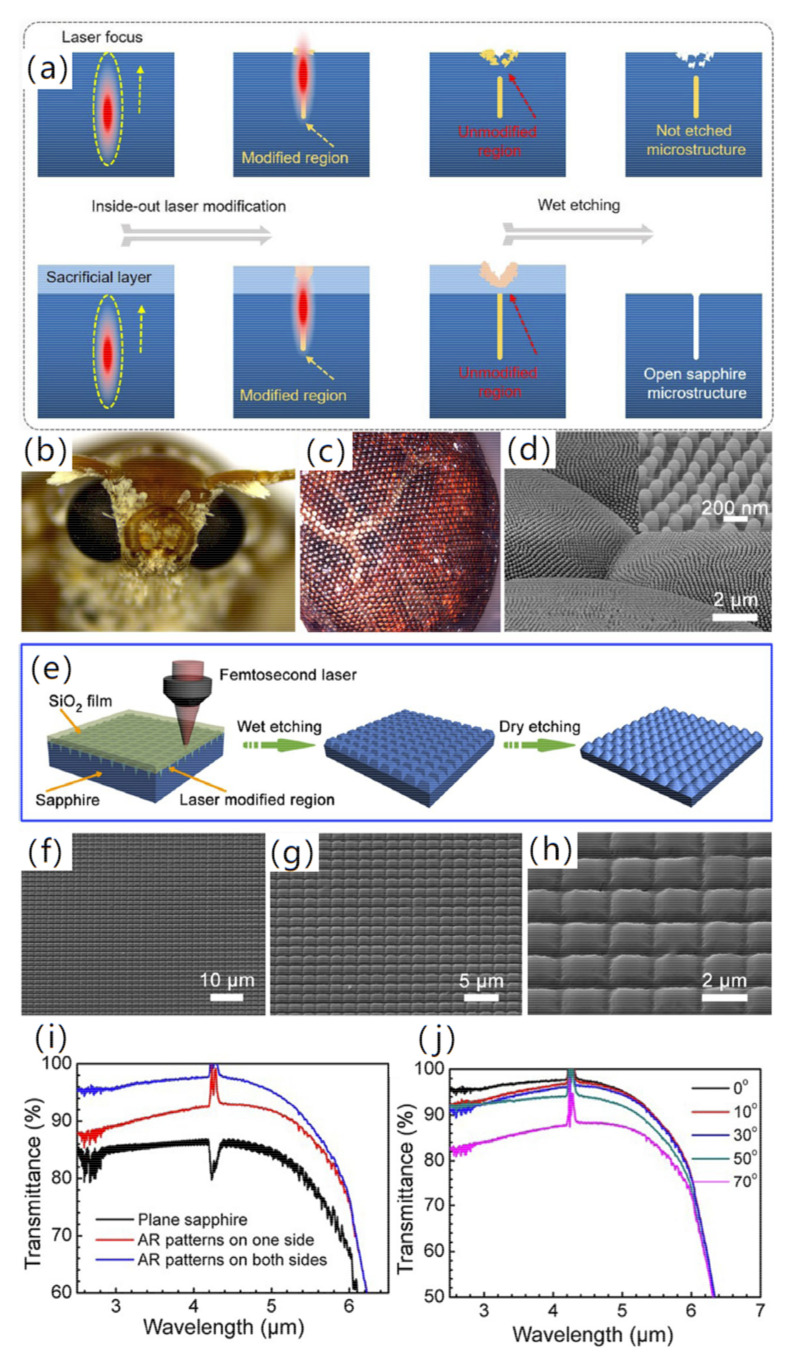
(**a**) The schematic diagram of femtosecond laser modification along with subsequent wet etching of sapphire with and without a sacrificial layer; (**b**) Optical photograph, (**c**) LSCM image and (**d**) local SEM image of the moth eye; (**e**) Schematic diagram of preparation process of anti-reflection sapphire surface for bionic moth eye; (**f**–**h**) SEM images of the bionic moth-eye structures on sapphire; (**i**) Experimentally measured transmittance of one-sided and two-sided processed sapphire in the mid-infrared band; (**j**) The relationship between transmittance and incident angle of sapphire with anti-reflection structures on both sides [75], Copyright © 2022 Springer Nature.

In 2017, Li et al. [74] proposed a strategy for wet etching-assisted femtosecond laser processing of sapphire and fabricated inverted pyramid and cone arrays with a period of about 2 μm and a height of about 900 nm on sapphire, which significantly inhibited the sapphire’s specular reflection in the 3–5 μm band. The transmittance reached a maximum of 92.5% at the wavelength of 4 μm. The prepared sub-wavelength array structure is consistent with the sapphire bulk material, which can overcome the mismatch between the coating and the thin film. In 2022, Liu et al. [75] proposed an inside-out femtosecond laser deep scribing technology in combination with an etching process for fabricating bio-inspired micro/nanostructures with a high aspect ratio on sapphire. By introducing a sacrificial layer protection strategy, the damage competition between the surface and the interior could be effectively avoided. It provides a new idea for the preparation of biomimetic micro/nanostructures. Based on this technology, a sapphire infrared anti-reflection window with a double-sided pyramid structure array of biomimetic moth-eyes is designed and fabricated, which achieves a significant improvement in mid-infrared broadband transmittance. The transmittance of the 3–5 μm band is higher than 95%. Especially at 4 μm, the transmittance is as high as 98%. In addition, the transmittance is higher than 95% in the range of the incident angle of 0–70°, as shown in Figure 5. The results show that the sacrificial layer-assisted femtosecond laser deep processing technology is an effective and versatile technology, which provides a broad prospect for the preparation of special micro-nano optical devices of hard brittle materials.

### 3.4. Diamond

Diamond is transparent in the visible, ultraviolet, and infrared regions, and possess a thermal conductivity for more than 20 W cm ^−1^ K ^−1^ at room temperature. In addition, it features chemical inertness, radiation stability, and biocompatibility [114], enabling its applications in ultra-high precision machining tools [115], microelectromechanical systems (MEMS) [116], sturdy optical gratings [117], high power lasers [118,119,120], and electronic devices suitable for harsh environments [121].

It is the most desirable material in the world, although best known as the “hardest to machine” material, as any conventional material removal processes (MRPs) cannot be effective at the nanometer or micrometer scale with acceptable precision and accuracy when processing diamond crystals. Many MRPs have already been used to machine diamonds, which includes electrical discharge [122,123] and abrasive water jet machining [124], mechanical grinding [125], and laser processing [126,127]. Among these methods, laser processing technology has been widely used and is the most successfully used MRP by far [127,128,129], since the proper selection of wavelength, pulse duration, and power can lead to high-quality, tailored material surfaces and volume machining with precision down to a few nanometers [130,131].

**Figure 6:**
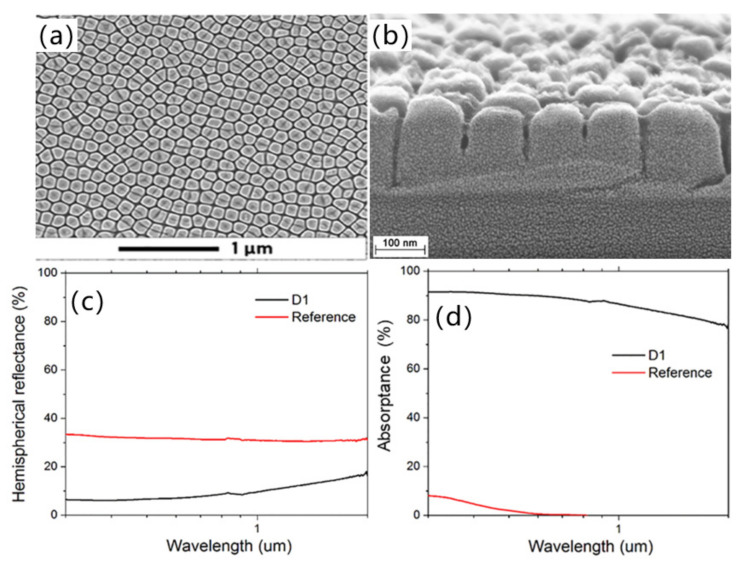
SEM images (**a**) top view and (**b**) cross-sectional view of a 2D laser-induced periodic surface structure with deep subwavelength periodicity on the diamond surface; (**c**) Reflectance spectrum of the diamond sample; (**d**) Absorptivity spectrum of the diamond sample [133], Copyright © 2021, American Chemical Society.

In 2017, Granados et al. [132] used a highly controllable direct femtosecond UV laser-induced periodic surface structure (LIPSS) to fabricate a photonic surface structure on a single crystal diamond and obtained a high-quality and high-fidelity surface roughness less than 1.4 nm. For the diamond grating structure, the simulation results showed that the surface structure with a period of 400 nm and a depth of 120 nm enhanced the transmittance of light with wavelengths greater than 1.2 μm. The maximum transmittance at a wavelength of 1.25 μm is close to 100%. This processing method can be employed as a simple alternative to fabricate moth-eye diamond anti-reflection coatings for the development of near-infrared high-power diamond Raman lasers. In 2021, Mastellone et al. [133] designed a Michelson-like interferometer structure to generate two time-delayed cross-polarized femtosecond laser pulse sequences with a deep subwavelength period (Λ ≈ λ/10 ≈ 80 nm) on the diamond surface for the first time. The 2D laser-induced periodic surface structure exhibited remarkable anti-reflection properties after removing surface debris by chemical etching, which increased the absorption rate of visible light by 50 times. As shown in Figure 6, it is demonstrated that the nanostructures 2D periodicity can be tuned by regulating the number of pulses illuminating on the surface. If the delay between the two pulses is ≤2 ps, it is a promising option for the large-scale fabrication of 2D-LIPSSs on diamond, which paves a way for the formation of metasurfaces on diamond-based optoelectronic devices in future.

## 4. Application of Hard Material Anti-Reflection Coating

Anti-reflection coatings (ARCs) are one of the most effective ways to suppress light reflection and promote light transmission and absorption. Generally, ARCs can be utilized to improve the efficiency of photovoltaic and optical devices. In this section, we will therefore briefly present the recent advances in the application of anti-reflection materials based on arrays of biomimetic structures.

### 4.1. Infrared Optical Window

Since infrared technology plays an essential role in the military field, it is necessary to develop infrared window materials. At present, the commonly used infrared window materials mainly include zinc sulfide, germanium, spinel, diamond, and sapphire. For window materials, the precise of instrument can be improved with higher transmittance. Compared with the traditional anti-reflection film, the fabrication of a sub-wavelength anti-reflection structure on the material can overcome the mismatch between the film and the material. In addition, the damage and peeling problems can be solved to improve the thermal stability, mechanical strength, and chemical stability. Li et al. [74] fabricated inverted pyramid and cone arrays with a period of about 2 μm with a height of about 900 nm on sapphire. Liu et al. [75] further designed and fabricated a bionic moth-eye sapphire infrared anti-reflection window, which significantly improved the mid-infrared anti-reflection performance of sapphire. Especially at the wavelength of 4 μm, its transmittance was as high as 98%; thus, replacing traditional coating anti-reflection technology with structural refractive index gradient provides an effective solution for long-term applications in practical harsh environments. This has essential technical significance for demanding optical windows in military mid-infrared devices.

### 4.2. Photodetectors

Photodetectors are capable of converting optical signals into electrical signals based on the photoconductive effect, which plays a vital role in infrared imaging and infrared remote sensing. The anti-reflection structure can increase light absorption of the photodetector to enhance the sensitivity of the photodetector. Li et al. [134] irradiated the silicon surface with a femtosecond laser under nitrogen, simultaneously doping N atoms into the structured silicon surface. It was found that the double absorber layer could improve the photoresponsivity to 5.3 mA/W under the same reverse bias compared to the single absorber layer. Photodetectors are developing rapidly in the direction of high speed, high sensitivity, and wide bandwidth, which is widely used in complex systems such as optical communication systems, signal processing devices, and sensing devices. It is believed that with the assistance of femtosecond laser processing technology, surfaces with anti-reflection properties such as wide bandwidth, whole angle, and high absorption will make the photodetector more sensitive.

Photothermal conversion is also a basic form of energy conversion. Effective collection and utilization of solar energy for photothermal conversion to drive desalination is a vital way to resolve the shortage of freshwater resources at present [135,136,137,138]. Among them, it is imperative to prepare photothermal layers with high light absorption rate and high photothermal conversion efficiency, which affect the rate of seawater desalination and the performance of the overall device [90,139]. Fan et al. [140] used femtosecond laser direct writing technology to fabricate a cauliflower structure on the copper surface with an absorption rate of 98% in the range of 200–800 nm, which was used as a photothermal conversion layer at 1 kW h^−1^ solar irradiation; the overall photothermal conversion efficiency was increased to more than 60%.

### 4.3. Multi-Functional Composite Surface

In practical applications, these devices with anti-reflection surfaces will inevitably encounter extreme conditions, such as foggy, rainy, dusty, and bacterial environments. This requires the anti-reflection surface to have self-cleaning, anti-fog, anti-icing, anti-bacterial, and anti-corrosion functions, which can improve the working stability of the device in harsh environments. Long-term outdoor work will lead to dust accumulation on the solar cells surface, which will seriously affect the absorption efficiency of sunlight, thereby affecting the performance of the equipment. The material surface with super-hydrophobic properties will take away the dust and other pollutants accumulated on the surface under the action of water droplets, thereby realizing the function of being self-cleaning. Mao et al. [141] successfully fabricated micro-protrusion arrays decorated with nanoneedles on the copper surface by femtosecond laser processing combined with thermal oxidation. The total reflectivity of the processed surface can be stably lower than 6% in a wide wavelength range of 600–1150 nm. The maximum contact angle is 161°, and the minimum sliding angle is less than 1.7°, confirming that the surface has good super-hydrophobicity and extremely low adhesion. For the optical windows of some special equipment and protective glasses working in special environments, the structured surface with water and fog resistance and anti-icing properties can reduce the adverse effects of harsh environments and improve the accuracy of the equipment. Domke et al. [142] used femtosecond laser processing technology prepared periodic patterns of rippled circles or rough holes on the glass surface and obtained a waterproof surface with excellent performance. Li et al. [143] prepared lattice and periodic stripe structures on the copper surface. The results showed that the processed surface significantly prolonged the freezing time and had a good anti-icing performance.

## 5. Conclusions and Outlook

Anti-reflection surfaces based on micro/nanostructures have excellent properties such as wide-angle, broad-spectrum, and polarization insensitivity. They have been widely utilized within solar cells, LEDs/OLEDs, and photodetectors. In this review, the principle of anti-reflection surface, the preparation of anti-reflection structure, the selection of anti-reflection material, and the application of anti-reflection surface are briefly introduced. Furthermore, the research progresses of anti-reflective surfaces on hard materials by femtosecond laser processing technology in recent years are summarized.

Currently, femtosecond laser processing of anti-reflection surfaces has achieved significant advances in a wide range of materials. With the gradually developed chemical etching and the introduction of sacrificial layers and other means to assist the femtosecond laser processing strategy, the processed surface roughness is getting lower, and the optical performance is improved. However, there are still some pressing issues that need to be addressed, as listed below.

(1) The low processing efficiency of femtosecond laser processing technology make it difficult to achieve rapid large-area preparation. Various process-assisted femtosecond laser micro-nano processing technology (e.g., bottom-up strategy, wet-corrosion, annealing processes, etc.) and spatial light modulation technology are expected to solve the above problems in future, providing more options for designing and preparing anti-reflection surfaces.
(2) The current microstructure types of anti-reflection surfaces (cones, pyramids, nano spikes, gratings, etc.) have certain limitations, and it is a considerable challenge to obtain inspiration from natural biological surface structures and apply them to surface engineering science.
(3) Most of the existing anti-reflection surfaces are confined to flat surfaces, and a considerable proportion of optical windows in practical applications are curved surfaces. How to fabricate anti-reflection surfaces on curved surfaces and maintain their wide-angle and wide-spectrum characteristics is also a huge challenge. The true three-dimensional processing capability of the femtosecond laser may provide a solution to this challenge, which requires more exploration in femtosecond laser micro-nano processing technology.
(4) The anti-reflection surface working in a harsh environment needs to have mechanical stability, thermal stability, chemical stability, super-oleophobic, super-hydrophobic, anti-corrosion, and other characteristics, while maintaining the surface anti-reflection properties. It will be a future development trend to select suitable materials and processing methods according to the actual needs and develop an anti-reflective surface with multiple functional integrations.

Although there are still some problems in the fabrication of anti-reflection surfaces by femtosecond laser processing, more excellent integrated anti-reflection surfaces will definitely be fabricated through an in-depth study of the anti-reflection surface principle, optimization of the anti-reflection surface structure, and combination with other preparation processes. This will significantly enhance the optical devices performance, which in turn promote the development of solar energy absorption and utilization, infrared imaging, optoelectronic devices, aerospace, and other fields.

## Figures and Tables

**Table 1:** Unique anti-reflection (AR) structures and composite functions on biological surfaces.

Biological Surfaces	AR Structures	AR Mechanism	Functions	Reference
Moth eye	Nano-nipple structures	Change mutation refraction index into a continuously graded refraction index	Anti-reflection, anti-fogging	[45]
Moth wing	Nano-pillar structures	-	Anti-reflection	[46]
Butterfly eye	-	-	Anti-reflection	[47]
Butterfly wing	Nano-pillar structures, nano-hole structures, and hierarchical structures (concave multilayer structures, quasi-honeycomb structures, parallel ridges, parallel-laminae structures, inclined ridge-lamellae structures)	Destructive interference, multiple refraction and continuous gradient refractive index ARC *	Anti-reflection, structural color, light-trapping, anti-fogging, self-cleaning, super-hydrophobicity, chemical sensing capability	[48,49,50,51,52,53,54,55,56]
Fly eye	Nano-nipple structures	Change mutation refraction index into a continuously graded refraction index	Anti-reflection, anti-fogging	[57,58,59]
Beetle eye	Maze-like nanostructures	-	Anti-reflection	[60]

* ARC: Anti-reflective coating.

**Table 2:** Summary of fabrication methods and optical properties of anti-reflection (AR) structures on hard materials.

Materials	AR Structure	Fabrication Technology	Advantage and Disadvantage	Reflection (%)	Transmittance (%)	Wavelength (nm)	Reference
Silicon	Nanopillar	Metal-assisted chemical etching	Simple operation and easy access to high-aspect-ratio nanostructures, but special equipment required	<0.1	-	250–1050	[66]
Silicon	Nanowire	Colloidal lithography + Plasma etching	Fast, simple, low-priced, time-efficient and high-throughput, but difficult to access high aspect ratio	<2	-	250–950	[67]
Silicon	Asymmetric nanowire	Top-down lithography combined with a dry etching	Simple, fast, and easily tuned, but easily damage the surface, also sophisticated and expensive equipment required	<5	-	300–1000	[68]
Silicon	Hierarchical structures	Laser interference lithography + Laser direct writing + Metal-assisted chemical etching	Fast, high aspect ratio structures applicable, easy to fine-tune surface morphology and size, but special equipment needed	<1	-	300–1200	[69]
Fused silica wafer	Nanocone	Interference lithography + Chemical vapor deposition	High aspect ratio nanostructures and weakly curved substrates applicable, contact free, and easy-controllable of the size, but special equipment and multiple expensive steps required	-	>98	250–1700	[70]
Au	Nanocone	Colloidal lithography + Oxygen plasma etching	Facile, fast, and structure parameters were easily controlled, but special equipment and multiple etching process required	<1	-	450–900	[71]
ZnO	Nanorod	Hydrothermal growth	Easy-controllable and cost-effective, but long reacting period and special equipment needed	1.2	76.1	400–800	[72]
Sapphire	Nanocone on hemispherical submicrometer gratings	Thermally dewetted metal nanoparticles + Inductively coupled plasma dry etching	Effective, simple, and easily controlled, but required additional thermal treatments and special equipment	-	90.7	300–1100	[73]
Sapphire	Inverted pyramid and cone arrays	Femtosecond laser direct writing assist with wet etching	The fast preparation process, high efficiency, mass production, green, high precision, strong controllability, but needed special equipment	-	92.5	3000–5000	[74]
Sapphire	Double-sided subwavelength pyramid array	The sacrificial layer assisted inside-out femtosecond laser deep scribing + Wet-etching	The fast preparation process, high efficiency, mass production, green, high precision, strong controllability, but needed special equipment	-	~ 98	3000–5000	[75]

## Data Availability

Not applicable.

## References

[1] Fan P., Zhong M. (2016). Progress on ultrafast laser fabricating metal surface micro-nano antireflection structures. Infrared Laser Eng..

[2] Li C.H., Zhao J.H., Yu X.Y., Chen Q.D., Feng J., Sun H.B. (2016). Fabrication of black silicon with thermostable infrared absorption by femtosecond laser. IEEE Photonics J..

[3] Liu Y.F., An M.H., Zhang X.L., Bi Y.G., Yin D., Zhang Y.F., Feng J., Sun H.B. (2016). Enhanced efficiency of organic light-emitting devices with corrugated nanostructures based on soft nano-imprinting lithography. Appl. Phys. Lett..

[4] Han D.D., Chen Z.D., Li J.C., Mao J.W., Jiao Z.Z., Wang W., Zhang W., Zhang Y.L., Sun H.B. (2020). Airflow enhanced solar evaporation based on janus graphene membranes with stable interfacial floatability. ACS Appl. Mater. Interfaces.

[5] Tan G.J., Lee J.H., Lan Y.H., Wei M.K., Peng L.H., Cheng I.C., Wu S.T. (2017). Broadband antireflection film with moth-eye-like structure for flexible display applications. Optica.

[6] Moghimi M.J., Lin G.Y., Jiang H.R. (2018). Broadband and ultrathin infrared stealth sheets. Adv. Eng. Mater..

[7] Yao L., He J.H., Geng Z., Ren T.T. (2015). Fabrication of mechanically robust, self-cleaning and optically high-performance hybrid thin films by SiO_2_&TiO_2_ double-shelled hollow nanospheres. Nanoscale.

[8] Kim D.S., Jeong Y., Jeong H., Jang J.H. (2014). Triple-junction InGaP/GaAs/Ge solar cells integrated with polymethyl methacrylate subwavelength structure. Appl. Surf. Sci..

[9] Raut H.K., Dinachali S.S., He A.Y., Ganesh V.A., Saifullah M.S.M., Law J., Ramakrishna S. (2013). Robust and durable polyhedral oligomeric silsesquioxane-based anti-reflective nanostructures with broadband quasi-omnidirectional properties. Energy Environ. Sci..

[10] Shi G., Guo J.L., Wang L.K., Sang X.X., Wang J., Yang J.G., Li Y. (2017). Photoactive PANI/TiO_2_/Si composite coatings with 3D bio-inspired structures. New J. Chem..

[11] Su W.X., Wu C.Y., Lee Y.C. (2019). Anti-reflection nano-structures fabricated on curved surface of glass lens based on metal contact printing lithography. Microelectron. Eng..

[12] Zhang Y.L., Chen Q.D., Xia H., Sun H.B. (2010). Designable 3D nanofabrication by femtosecond laser direct writing. Nano Today.

[13] Zhang Y.L., Guo L., Wei S., He Y.Y., Xia H., Chen Q.D., Sun H.B., Xiao F.S. (2010). Direct imprinting of microcircuits on graphene oxides film by femtosecond laser reduction. Nano Today.

[14] Zhang Y.L., Tian Y., Wang H., Ma Z.C., Han D.D., Niu L.G., Chen Q.D., Sun H.B. (2019). Dual-3D femtosecond laser nanofabrication enables dynamic actuation. ACS Nano.

[15] Fang H.H., Yang J., Ding R., Chen Q.D., Wang L., Xia H., Feng J., Ma Y.G., Sun H.B. (2010). Polarization dependent two-photon properties in an organic crystal. Appl. Phys. Lett..

[16] Liu Y.Q., Mao J.W., Chen Z.D., Han D.D., Jiao Z.Z., Ma J.N., Jiang H.B., Yang H. (2020). Three-dimensional micropatterning of graphene by femtosecond laser direct writing technology. Opt. Lett..

[17] You R., Liu Y.Q., Hao Y.L., Han D.D., Zhang Y.L., You Z. (2020). Laser fabrication of graphene-based flexible electronics. Adv. Mater..

[18] You R., Han D.D., Liu F.M., Zhang Y.L., Lu G.Y. (2018). Fabrication of flexible room-temperature NO_2_ sensors by direct laser writing of In_2_O_3_ and graphene oxide composites. Sens. Actuators B Chem..

[19] Jiang H.B., Liu Y., Liu J., Li S.Y., Song Y.Y., Han D.D., Ran L.Q. (2019). Moisture-responsive graphene actuators prepared by two-beam laser interference of graphene oxide paper. Front. Chem..

[20] Zhang D.S., Ranjan B., Tanaka T., Sugioka K. (2020). Carbonized hybrid micro/nanostructured metasurfaces produced by femtosecond laser ablation in organic solvents for biomimetic antireflective surfaces. ACS Appl. Nano Mater..

[21] Ning J.Q., Sievers D.E., Garmestani H., Liang S.Y. (2020). Analytical modeling of in-process temperature in powder feed metal additive manufacturing considering heat transfer boundary condition. Int. J. Precis. Eng. Manuf.-Green Technol..

[22] Ning J.Q., Sievers D.E., Garmestani H., Liang S.Y. (2019). Analytical modeling of in-process temperature in powder bed additive manufacturing considering laser power absorption, latent heat, scanning strategy, and powder packing. Materials.

[23] Vorobyev A.Y., Guo C.L. (2011). Antireflection effect of femtosecond laser-induced periodic surface structures on silicon. Opt. Express.

[24] Fu X.Y., Chen Z.D., Han D.D., Zhang Y.L., Xia H., Sun H.B. (2020). Laser fabrication of graphene-based supercapacitors. Photonics Res..

[25] Liu Y.Q., Chen Z.D., Mao J.W., Han D.D., Sun X. (2019). Laser fabrication of graphene-based electronic skin. Front. Chem..

[26] Fang H.H., Ding R., Lu S.Y., Yang J., Zhang X.L., Yang R., Feng J., Chen Q.D., Song J.F., Sun H.B. (2012). Distributed feedback lasers based on thiophene/phenylene Co-Oligomer single crystals. Adv. Funct. Mater..

[27] Zou T., Zhao B., Xin W., Wang Y., Wang B., Zheng X., Xie H., Zhang Z., Yang J., Guo C. (2020). High-speed femtosecond laser plasmonic lithography and reduction of graphene oxide for anisotropic photoresponse. Light Sci. Appl..

[28] Sakakura M., Lei Y., Wang L., Yu Y.H., Kazansky P.G. (2020). Ultralow-loss geometric phase and polarization shaping by ultrafast laser writing in silica glass. Light Sci. Appl..

[29] Zhao Z.Y., Song Z.Q., Shi W.Z., Zhao Q.Z. (2014). Optical absorption and photocurrent enhancement in semi-insulating gallium arsenide by femtosecond laser pulse surface microstructuring. Opt. Express.

[30] Cheng Y. (2017). Internal Laser writing of high-aspect-ratio microfluidic structures in silicate glasses for lab-on-a-chip applications. Micromachines.

[31] Scott S., Ali Z. (2021). Fabrication methods for microfluidic devices: An overview. Micromachines.

[32] Butkute A., Jonusauskas L. (2021). 3D manufacturing of glass microstructures using femtosecond laser. Micromachines.

[33] Hazzan K.E., Pacella M., See T.L. (2021). Laser processing of hard and ultra-hard materials for micro-machining and surface engineering applications. Micromachines.

[34] Wang L., Jiao L., Pang S.S., Yan P., Wang X.B., Qiu T.Y. (2021). The development of design and manufacture techniques for bioresorbable coronary artery stents. Micromachines.

[35] Liu X.Q., Bai B.F., Chen Q.D., Sun H.B. (2019). Etching-assisted femtosecond laser modification of hard materials. Opto-Electron. Adv..

[36] Sima F., Xu J., Wu D., Sugioka K. (2017). Ultrafast laser fabrication of functional biochips: New avenues for exploring 3D micro-and nano-environments. Micromachines.

[37] Cao X.W., Chen Q.D., Fan H., Zhang L., Juodkazis S., Sun H.B. (2018). Liquid-assisted femtosecond laser precision-machining of silica. Nanomaterials.

[38] Wang D., Li Y.G., Zhang C.C., Liao W., Li Z.Y., Zhang Q.H., Xu Q. (2019). Broadband terahertz antireflective microstructures on quartz crystal surface by CO_2_ laser micro-processing. Opt. Express.

[39] Boden S.A., Bagnall D.M. (2008). Tunable reflection minima of nanostructured antireflective surfaces. Appl. Phys. Lett..

[40] Brunner R., Sandfuchs O., Pacholski C., Morhard C., Spatz J. (2012). Lessons from nature: Biomimetic subwavelength structures for high-performance optics. Laser Photonics Rev..

[41] Kubota S., Kanomata K., Ahmmad B., Mizuno J., Hirose F. (2016). Optimized design of moth eye antireflection structure for organic photovoltaics. J. Coat. Technol. Res..

[42] Han Z.W., Jiao Z.B., Niu S.C., Ren L.Q. (2019). Ascendant bioinspired antireflective materials: Opportunities and challenges coexist. Prog. Mater. Sci..

[43] Ghymn Y.H., Jung K., Shin M., Ko H. (2015). A luminescent down-shifting and moth-eyed anti-reflective film for highly efficient photovoltaic devices. Nanoscale.

[44] Ji S., Park J., Lim H. (2012). Improved antireflection properties of moth eye mimicking nanopillars on transparent glass: Flat antireflection and color tuning. Nanoscale.

[45] Li J., Zhu J., Gao X.F. (2014). Bio-inspired high-performance antireflection and antifogging polymer films. Small.

[46] Deparis O., Khuzayim N., Parker A., Vigneron J.P. (2009). Assessment of the antireflection property of moth wings by three-dimensional transfer-matrix optical simulations. Phys. Rev. E.

[47] Stavenga D.G., Foletti S., Palasantzas G., Arikawa K. (2006). Light on the moth-eye corneal nipple array of butterflies. Proc. R. Soc. B.

[48] Wang W.L., Zhang W., Fang X.T., Huang Y.Q., Liu Q.L., Bai M.W., Zhang D. (2014). Omnidirectional light absorption of disordered nano-hole structure inspired from Papilio ulysses. Opt. Lett..

[49] Zhang W., Gu J.J., Liu Q.L., Su H.L., Fan T.X., Zhang D. (2014). Butterfly effects: Novel functional materials inspired from the wings scales. Phys. Chem. Chem. Phys..

[50] Hang Z.W., Mu Z.Z., Li B., Wang Z., Zhang J.Q., Niu S.C., Ren L.Q. (2016). Active antifogging property of monolayer SiO_2_ film with bioinspired multiscale hierarchical pagoda structures. ACS Nano.

[51] Liu C.C., Ju J., Zheng Y.M., Jiang L. (2014). Asymmetric ratchet effect for directional transport of fog drops on static and dynamic butterfly wings. ACS Nano.

[52] Zyla G., Kovalev A., Grafen M., Gurevich E.L., Esen C., Ostendorf A., Gorb S. (2017). Generation of bioinspired structural colors via two-photon polymerization. Sci. Rep..

[53] Han Z., Fu J., Wang Z., Wang Y.G., Li B., Mu Z.Z., Zhang J.Q., Niu S.C. (2017). Long-term durability of superhydrophobic properties of butterfly wing scales after continuous contact with water. Colloids Surf. A Physicochem. Eng. Asp..

[54] Han Z.W., Niu S.C., Yang M., Mu Z.Z., Li B., Zhang J.Q., Ye J.F., Ren L.Q. (2014). Unparalleled sensitivity of photonic structures in butterfly wings. RSC Adv..

[55] Niu S.C., Li B., Mu Z.Z., Yang M., Zhang J.Q., Han Z.W., Ren L.Q. (2015). Excellent structure-based multifunction of morpho butterfly wings: A review. J. Bionic Eng..

[56] Han Z.W., Yang M., Li B., Mu Z.Z., Niu S.C., Zhang J.Q., Yang X. (2016). Excellent color sensitivity of butterfly wing scales to liquid mediums. J. Bionic Eng..

[57] Kryuchkov M., Katanaev V.L., Enin G.A., Sergeev A., Timchenko A.A., Serdyuk I.N. (2011). Analysis of micro-and nano-structures of the corneal surface of drosophila and its mutants by atomic force microscopy and optical diffraction. PLoS ONE.

[58] Sun Z.Q., Liao T., Liu K.S., Jiang L., Kim J.H., Dou S.X. (2014). Fly-eye inspired superhydrophobic anti-fogging inorganic nanostructures. Small.

[59] Huang J.Y., Wang X.D., Wang Z.L. (2008). Bio-inspired fabrication of antireflection nanostructures by replicating fly eyes. Nanotechnology.

[60] Blagodatski A., Kryuchkov M., Sergeev A., Klimov A.A., Shcherbakov M.R., Enin G.A., Katanaev V.L. (2014). Under- and over-water halves of Gyrinidae beetle eyes harbor different corneal nanocoatings providing adaptation to the water and air environments. Sci. Rep..

[61] Zhang Y.B., Yuan M.H., Chen L., Cai B., Yang R., Zhu Y.M. (2016). Broadband terahertz anti-reflective structure fabricated by femtosecond laser drilling technique. Opt. Commun..

[62] Corrigan T.D., Park D.H., Drew H.D., Guo S.H., Kolb P.W., Herman W.N., Phaneuf R.J. (2012). Broadband and mid-infrared absorber based on dielectric-thin metal film multilayers. Appl. Opt..

[63] Yang Q.R., He S., Huang R.M., Yu M., Chen C., Zheng S.S., Yun D.Q., Zheng L.L., Cheng Q.J. (2021). Research on the fabrication and anti-reflection performance of diamond-like carbon films. Diamond Relat. Mater..

[64] Kumar A., Yerva S.V., Barshilia H.C. (2016). Broadband and wide angle anti-reflective nanoporous surface on poly (ethylene terephthalate) substrate using a single step plasma etching for applications in flexible electronics. Sol. Energy Mater. Sol. Cells.

[65] Kim S., Jung U.T., Kim S.K., Lee J.H., Choi H.S., Kim C.S., Jeong M.Y. (2015). Nanostructured multifunctional surface with antireflective and antimicrobial characteristics. ACS Appl. Mater. Interfaces.

[66] Teng F., Li N., Liu L.X., Xu D.R., Xiao D.Y., Lu N. (2016). Fabrication of ordered Si nanopillar arrays for ultralow reflectivity. RSC Adv..

[67] Smyrnakis A., Almpanis E., Constantoudis V., Papanikolaou N., Gogolides E. (2015). Optical properties of high aspect ratio plasma etched silicon nanowires: Fabrication-induced variability dramatically reduces reflectance. Nanotechnology.

[68] Ko M.D., Rim T., Kim K., Meyyappan M., Baek C.K. (2015). High efficiency silicon solar cell based on asymmetric nanowire. Sci. Rep..

[69] Yang J., Luo F.F., Kao T.S., Li X., Ho G.W., Teng J.H., Luo X.G., Hong M.H. (2014). Design and fabrication of broadband ultralow reflectivity black Si surfaces by laser micro/nanoprocessing. Light Sci. Appl..

[70] Park K.C., Choi H.J., Chang C.H., Cohen R.E., McKinley G.H., Barbastathis G. (2012). Nanotextured silica surfaces with robust superhydrophobicity and omnidirectional broadband supertransmissivity. ACS Nano.

[71] Toma M., Loget G., Corn R.M. (2013). Fabrication of broadband antireflective plasmonic gold nanocone arrays on flexible polymer films. Nano Lett..

[72] Li B.J., Huang L.J., Ren N.F., Kong X., Cai Y.L., Zhang J.L. (2016). Superhydrophobic and anti-reflective ZnO nanorod-coated FTO transparent conductive thin films prepared by a three-step method. J. Alloys Compd..

[73] Leem J.W., Kim M.S., Yu J.S. (2013). Broadband highly transparent sapphires with biomimetic antireflective compound submicrometer structures for optical and optoelectronic applications. J. Opt. Soc. Am. B.

[74] Li Q.K., Cao J.J., Yu Y.H., Wang L., Sun Y.L., Chen Q.D., Sun H.B. (2017). Fabrication of an anti-reflective microstructure on sapphire by femtosecond laser direct writing. Opt. Lett..

[75] Liu X.Q., Zhang Y.L., Li Q.K., Zheng J.X., Lu Y.M., Juodkazis S., Chen Q.D., Sun H.B. (2022). Biomimetic sapphire windows enabled by inside-out femtosecond laser deep-scribing. PhotoniX.

[76] Li Z.Z., Wang L., Fan H., Yu Y.H., Chen Q.D., Juodkazis S., Sun H.B. (2020). O-FIB: Far-field-induced near-field breakdown for direct nanowriting in an atmospheric environment. Light Sci. Appl..

[77] Wang A., Jiang L., Li X., Liu Y., Dong X., Qu L., Duan X., Lu Y. (2015). Mask-free patterning of high-conductivity metal nanowires in open air by spatially modulated femtosecond laser pulses. Adv. Mater..

[78] Wang S.J., Jiang L., Han W.N., Liu W., Hu J., Wang S.C., Lu Y.F. (2020). Controllable formation of laser-induced periodic surface structures on ZnO film by temporally shaped femtosecond laser scanning. Opt. Lett..

[79] Huang J., Jiang L., Li X.W., Wei Q.S., Wang Z.P., Li B.H., Huang L.L., Wang A.D., Wang Z., Li M. (2019). Cylindrically focused nonablative femtosecond laser processing of long-range uniform periodic surface structures with tunable diffraction efficiency. Adv. Opt. Mater..

[80] Qiao M., Wang H.M., Lu H.J., Li S., Yan J.F., Qu L.T., Zhang Y.Y., Jiang L., Lu Y.F. (2020). Micro/nano processing of natural silk fibers with near-field enhanced ultrafast laser. Sci. China Mater..

[81] Zuo P., Jiang L., Li X., Tian M.Y., Xu C.Y., Yuan Y.J., Ran P., Li B., Lu Y.F. (2019). Maskless micro/nanopatterning and bipolar electrical rectification of MoS_2_ flakes through femtosecond laser direct writing. ACS Appl. Mater. Interfaces.

[82] Zhang Y.C., Li Y., Hu Y.L., Zhu X.L., Huang Y.W., Zhang Z., Rao S.L., Hu Z.J., Qiu W.X., Wang Y.L. (2018). Localized self-growth of reconfigurable architectures induced by a femtosecond laser on a shape-memory polymer. Adv. Mater..

[83] Lao Z.X., Xia N., Wang S.J., Xu T.T., Wu X.Y., Zhang L. (2021). Tethered and untethered 3D microactuators fabricated by two-photon polymerization: A review. Micromachines.

[84] Gao S., Li Z.Z., Hu Z.Y., Yu F., Chen Q.D., Tian Z.N., Sun H.B. (2020). Diamond optical vortex generator processed by ultraviolet femtosecond laser. Opt. Lett..

[85] Huang J., Jiang L., Li X.W., Wang A.D., Wang Z., Wang Q.S., Hu J., Qu L.T., Cui T.H., Lu Y.F. (2019). Fabrication of highly homogeneous and controllable nanogratings on silicon via chemical etching-assisted femtosecond laser modification. Nanophotonics.

[86] Liu X.Q., Yu L., Yang S.N., Chen Q.D., Wang L., Juodkazis S., Sun H.B. (2019). Optical nanofabrication of concave microlens arrays. Laser Photonics Rev..

[87] Yu H.W., Li X., Hao Z.Q., Xiong W., Guo L.B., Lu Y.F., Yi R.X., Li J.M., Yang X.Y., Zeng X.Y. (2017). Fabrication of metal/semiconductor nanocomposites by selective laser nano-welding. Nanoscale.

[88] Rodenas A., Gu M., Corrielli G., Paie P., John S., Kar A.K., Osellame R. (2019). Three-dimensional femtosecond laser nanolithography of crystals. Nat. Photonics.

[89] Chen T., Wang W.J., Tao T., Pan A.F., Mei X.S. (2020). Multi-scale micro-nano structures prepared by laser cleaning assisted laser ablation for broadband ultralow reflectivity silicon surfaces in ambient air. Appl. Surf. Sci..

[90] Guo C.F., Sun T.Y., Cao F., Liu Q., Ren Z.F. (2014). Metallic nanostructures for light trapping in energy-harvesting devices. Light Sci. Appl..

[91] Xiong X., Jiang S.C., Hu Y.H., Peng R.W., Wang M. (2013). Structured metal film as a perfect absorber. Adv. Mater..

[92] Qin Y.S., Zhang M.J., Guan Y., Huang X.G. (2019). Laser absorption and infrared stealth properties of Al/ATO composites. Ceram. Int..

[93] Kim J., Han K., Hahn J.W. (2017). Selective dual-band metamaterial perfect absorber for infrared stealth technology. Sci. Rep..

[94] Kodiyath R., Malak S.T., Combs Z.A., Koenig T., Mahmoud M.A., El-Sayed M.A., Tsukruk V.V. (2013). Assemblies of silver nanocubes for highly sensitive SERS chemical vapor detection. J. Mater. Chem. A.

[95] Gao C.B., Lu Z.D., Liu Y., Zhang Q., Chi M.F., Cheng Q., Yin Y.D. (2012). Highly stable silver nanoplates for surface plasmon resonance biosensing. Angew. Chem. Int. Ed..

[96] Fan P.X., Bai B.F., Zhong M.L., Zhang H.J., Long J.Y., Han J.P., Wang W.Q., Jin G.F. (2017). General strategy toward dual-scale-controlled metallic micro-nano hybrid structures with ultralow reflectance. ACS Nano.

[97] Kats M.A., Blanchard R., Genevet P., Capasso F. (2013). Nanometre optical coatings based on strong interference effects in highly absorbing media. Nat. Mater..

[98] Rajab F.H., Whitehead D., Liu Z., Li L. (2017). Characteristics of hierarchical micro/nano surface structure formation generated by picosecond laser processing in water and air. Appl. Phys. B Lasers Opt..

[99] Tan X., Tao Z., Yu M.X., Wu H.X., Li H.W. (2018). Anti-reflectance investigation of a micro-nano hybrid structure fabricated by dry/wet etching methods. Sci. Rep..

[100] Aydin K., Ferry V.E., Briggs R.M., Atwater H.A. (2011). Broadband polarization-independent resonant light absorption using ultrathin plasmonic super absorbers. Nat. Commun..

[101] Vorobyev A.Y., Guo C.L. (2013). Direct femtosecond laser surface nano/microstructuring and its applications. Laser Photonics Rev..

[102] Yao C.Z., Ye Y.Y., Jia B.S., Li Y., Ding R.J., Jiang Y., Wang Y.X., Yuan X.D. (2017). Polarization and fluence effects in femtosecond laser induced micro/nano structures on stainless steel with antireflection property. Appl. Surf. Sci..

[103] Xu S.Z., Tan L., Yao C.Z., Miao X.X., Lu H.B., Jiang X.D., Yuan X.D. (2021). Anti-reflective and wetting properties of femtosecond pulsed laser textured Al alloy surfaces. Optik.

[104] Fan P.X., Bai B.F., Jin G.F., Zhang H.J., Zhong M.L. (2018). Patternable fabrication of hyper-hierarchical metal surface structures for ultrabroadband antireflection and self-cleaning. Appl. Surf. Sci..

[105] Liu H.L., Hu J., Jiang L., Zhan S.H., Ma Y.L., Xu Z.J., Lu Y.F. (2020). Ultrabroad antireflection urchin-like array through synergy of inverse fabrications by femtosecond laser-tuned chemical process. Appl. Surf. Sci..

[106] Muslimov A.E., Asadchikov V.E., Butashin A.V., Vlasov V.P., Deryabin A.N., Roshchin B.S., Sulyanov S.N., Kanevsky V.M. (2016). Supersmooth and modified surface of sapphire crystals: Formation, characterization, and applications in nanotechnologies. Crystallogr. Rep..

[107] Li Q.K., Yu Y.H., Wang L., Cao X.W., Liu X.Q., Sun Y.L., Chen Q.D., Duan J.A., Sun H.B. (2016). Sapphire-based Fresnel zone plate fabricated by femtosecond laser direct writing and wet etching. IEEE Photonics Technol. Lett..

[108] Li Q.K., Chen Q.D., Niu L.G., Yu Y.H., Wang L., Sun Y.L., Sun H.B. (2016). Sapphire-based Dammann gratings for UV beam splitting. IEEE Photonics J..

[109] Tian Z.N., Hua J.G., Yu F., Yu Y.H., Liu H., Chen Q.D., Sun H.B. (2018). Aplanatic zone plate embedded in sapphire. IEEE Photonics Technol. Lett..

[110] Hua J.G., Hu Z.Y., Xu S.J., Tian Z.N., Yu Y.H., Chen Q.D., Sun H.B. (2019). Centimeter-sized aplanatic hybrid diffractive-refractive lens. IEEE Photonics Technol. Lett..

[111] Liu X.Q., Yang S.N., Yu L., Chen Q.D., Zhang Y.L., Sun H.B. (2019). Rapid engraving of artificial compound eyes from curved sapphire substrate. Adv. Funct. Mater..

[112] Lu Y.M., Tian Z.N., Yang S.N., Hua J.G., Liu X.Q., Zhao Y., Chen Q.D., Zhang Y.L., Sun H.B. (2019). High-efficiency spiral zone plates in sapphire. IEEE Photonics Technol. Lett..

[113] Takaku R., Hanany S., Imada H., Ishino H., Katayama N., Komatsu K., Konishi K., Kuwata-Gonokami M., Matsumura T., Mitsuda K. (2020). Broadband, millimeter-wave anti-reflective structures on sapphire ablated with femto-second laser. J. Appl. Phys..

[114] Jeong B., Lee B., Kim J.H., Choi J.A., Yang J., Sall E.G., Kim J.W., Heo D., Jang J., Kim G.H. (2020). Drilling of sub-100 μm hourglass-shaped holes in diamond with femtosecond laser pulses. Quantum Electron..

[115] Meier A. (2015). Diamond turning of diffractive microstructures. Precis. Eng..

[116] Cao Z.L., Aslam D. (2010). Fabrication technology for single-material MEMS using polycrystalline diamond. Diam. Relat. Mater..

[117] Forsberg P., Karlsson M. (2013). High aspect ratio optical gratings in diamond. Diam. Relat. Mater..

[118] Chen H., Bai Z., Yang X., Ding J., Qi Y., Yan B., Wang Y., Lu Z., Mildren R.P. (2022). Enhanced stimulated Brillouin scattering utilizing Raman conversion in diamond. Appl. Phys. Lett..

[119] Bai Z., Zhang Z., Wang K., Gao J., Zhang Z., Yang X., Wang Y., Lu Z., Mildren R.P. (2021). Comprehensive Thermal Analysis of Diamond in a High-Power Raman Cavity Based on FVM-FEM Coupled Method. Nanomaterials.

[120] Williams R.J., Kitzler O., Bai Z., Sarang S., Jasbeer H., McKay A., Antipov S., Sabella A., Lux O., Spence D.J. (2018). High Power Diamond Raman Lasers. IEEE J. Sel. Top. Quantum Electron..

[121] Wort C.J.H., Balmer R.S. (2008). Diamond as an electronic material. Mater. Today.

[122] Morgan C.J., Vallance R.R., Marsh E.R. (2004). Micro machining glass with polycrystalline diamond tools shaped by micro electro discharge machining. J. Micromech. Microeng..

[123] Wang D., Zhao W.S., Gu L., Kang X.M. (2011). A study on micro-hole machining of polycrystalline diamond by micro-electrical discharge machining. J. Mater. Process. Technol..

[124] Axinte D.A., Srinivasu D.S., Kong M.C., Butler-Smith P.W. (2009). Abrasive waterjet cutting of polycrystalline diamond: A preliminary investigation. Int. J. Mach. Tool Manu..

[125] Wu L.Q., Zhang H.J., Zong W.J., Du K. (2022). A theoretical model to predict the anisotropic characteristics in grinding of diamond conical indenter. J. Mater. Process. Technol..

[126] Dold C., Henerichs M., Gilgen P., Wegener K. (2013). Laser processing of coarse grain polycrystalline diamond (PCD) cutting tool inserts using picosecond laser pulses. Phys. Procedia.

[127] Ohfuji H., Okuchi T., Odake S., Kagi H., Sumiya H., Irifune T. (2010). Micro-/nanostructural investigation of laser-cut surfaces of single- and polycrystalline diamonds. Diam. Relat. Mater..

[128] Balling P., Schou J. (2013). Femtosecond-laser ablation dynamics of dielectrics: Basics and applications for thin films. Rep. Prog. Phys..

[129] Konov V.I. (2012). Laser in micro and nanoprocessing of diamond materials. Laser Photonics Rev..

[130] Gattass R.R., Mazur E. (2008). Femtosecond laser micromachining in transparent materials. Nat. Photonics.

[131] Bhuyan M.K., Courvoisier F., Lacourt P.A., Jacquot M., Salut R., Furfaro L., Dudley J.M. (2010). High aspect ratio nanochannel machining using single shot femtosecond Bessel beams. Appl. Phys. Lett..

[132] Granados E., Martinez-Calderon M., Gomez M., Rodriguez A., Olaizola S.M. (2017). Photonic structures in diamond based on femtosecond UV laser induced periodic surface structuring (LIPSS). Opt. Express.

[133] Mastellone M., Bellucci A., Girolami M., Serpente V., Polini R., Orlando S., Santagata A., Sani E., Hitzel F., Trucchi D.M. (2021). Deep-subwavelength 2D periodic surface nanostructures on diamond by double-pulse femtosecond laser irradiation. Nano Lett..

[134] Li C.H., Wang X.P., Zhao J.H., Zhang D.Z., Yu X.Y., Li X.B., Feng J., Chen Q.D., Ruan S.P., Sun H.B. (2018). Black silicon IR photodiode supersaturated with nitrogen by femtosecond laser irradiation. IEEE Sens. J..

[135] Li J.L., Wang X.Y., Lin Z.H., Xu N., Li X.Q., Liang J., Zhao W., Lin R.X., Zhu B., Liu G.L. (2020). Over 10 kg m^−2^ h^−1^ evaporation rate enabled by a 3D interconnected porous carbon foam. Joule.

[136] Li W.G., Li Z., Bertelsmann K., Fan D.E. (2019). Portable low-pressure solar steaming-collection unisystem with polypyrrole origamis. Adv. Mater..

[137] Liu F.H., Zhao B.Y., Wu W.P., Yang H.Y., Ning Y.S., Lai Y.J., Bradley R. (2018). Low cost, robust, environmentally friendly geopolymer-mesoporous carbon composites for efficient solar powered steam generation. Adv. Funct. Mater..

[138] Ren H.Y., Tang M., Guan B.L., Wang K.X., Yang J.W., Wang F.F., Wang M.Z., Shan J.Y., Chen Z.L., Wei D. (2017). Hierarchical graphene foam for efficient omnidirectional solar-thermal energy conversion. Adv. Mater..

[139] Jalil S.A., Lai B., ElKabbash M., Zhang J.H., Garcell E.M., Singh S., Guo C.L. (2020). Spectral absorption control of femtosecond laser-treated metals and application in solar-thermal devices. Light Sci. Appl..

[140] Fan P.X., Wu H., Zhong M.L., Zhang H.J., Bai B.F., Jin G.F. (2016). Large-scale cauliflower-shaped hierarchical copper nanostructures for efficient photothermal conversion. Nanoscale.

[141] Mao Z.W., Cao W., Hu J., Jiang L., Wang A.D., Li X., Cao J., Lu Y.F. (2017). A dual-functional surface with hierarchical micro/nanostructure arrays for self-cleaning and antireflection. RSC Adv..

[142] Domke M., Sonderegger G., Kostal E., Matylitsky V., Stroj S. (2019). Transparent laser-structured glasses with superhydrophilic properties for anti-fogging applications. Appl. Phys. A Mater. Sci. Process..

[143] Li J., Zhou Y.J., Wang W.B., Xu C.Y., Ren L.Q. (2020). Superhydrophobic copper surface textured by laser for delayed icing phenomenon. Langmuir.

